# Targeted delivery of engineered auditory sensing protein for ultrasound neuromodulation in the brain

**DOI:** 10.7150/thno.39786

**Published:** 2020-02-18

**Authors:** Chun-Yao Wu, Ching-Hsiang Fan, Nai-Hua Chiu, Yi-Ju Ho, Yu-Chun Lin, Chih-Kuang Yeh

**Affiliations:** 1 Department of Biomedical Engineering and Environmental Sciences, National Tsing Hua University, Hsinchu, Taiwan; 2 Institute of Nuclear Engineering and Sciences, National Tsing Hua University, Hsinchu, Taiwan; 3 Institute of Molecular Medicine, National Tsing Hua University, Hsinchu, Taiwan; 4 Department of Molecular Medicine, National Tsing Hua University, Hsinchu, Taiwan

**Keywords:** sonogenetic, neuromodulation, ultrasound, Prestin, microbubbles

## Abstract

Sonogenetics is a promising approach for *in vivo* neuromodulation using ultrasound (US) to non-invasively stimulate cells in deep tissue. However, sonogenetics requires accurate transduction of US-responsive proteins into target cells. Here, we introduce a non-invasive and non-viral approach for intracerebral gene delivery. This approach utilizes temporary ultrasonic disruption of the blood-brain barrier (BBB) to transfect neurons at specific sites in the brain via DNA that encodes engineered US-responsive protein (murine Prestin (N7T, N308S))-loaded microbubbles (pPrestin-MBs). Prestin is a transmembrane protein that exists in the mammalian auditory system and functions as an electromechanical transducer. We further improved the US sensitivity of Prestin by introducing specific amino acid substitutions that frequently occur in sonar species into the mouse Prestin protein. We demonstrated this concept in mice using US with pPrestin-MBs to non-invasively modify and activate neurons within the brain for spatiotemporal neuromodulation.

**Method:** MBs composed of cationic phospholipid and C_3_F_8_ loaded with mouse Prestin plasmid (pPrestin) via electrostatic interactions. The mean concentration and size of the pPrestin-MBs were (16.0 ± 0.2) × 10^9^ MBs/mL and 1.1 ± 0.2 μm, respectively. SH-SY5Y neuron-like cells and C57BL mice were used in this study. We evaluated the gene transfection efficiency and BBB-opening region resulting from pPrestin-MBs with 1-MHz US (pressure = 0.1-0.5 MPa, cycle = 50-10000, pulse repetition frequency (PRF): 0.5-5 Hz, sonication time = 60 s) using green fluorescence protein (Venus) and Evans blue staining.

**Results:** The maximum pPrestin expression with the highest cell viability occurred at a pressure of 0.5 MPa, cycle number of 5000, and PRF of 1 Hz. The cellular transfection rate with pPrestin-MBs and US was 20.2 ± 2.5%, which was 1.5-fold higher than that of commercial transfection agents (LT-1). *In vivo* data suggested that the most profound expression of pPrestin occurred at 2 days after performing pPrestin-MBs with US (0.5 MPa, 240 s sonication time). In addition, no server erythrocyte extravasations and apoptosis cells were observed at US-sonicated region. We further found that with 0.5-MHz US stimulation, cells with Prestin expression were 6-fold more likely to exhibit c-Fos staining than cells without Prestin expression.

**Conclusion:** Successful activation of Prestin-expressing neurons suggests that this technology provides non-invasive and spatially precise selective modulation of one or multiple specific brain regions.

## Introduction

Neurological disorders affect over 35% of the adult population and often include disorders of neural circuits characterized by specific spatial regions 1. Nevertheless, traditional medicine-based therapies for such diseases act throughout the brain, potentially affecting healthy areas 2, 3. Although surgery enables the targeting of specific sites in the brain for electrical stimulation or excision, it is associated with significant risks, such as tissue or neuronal damage 4. Existing treatments based on cellular or gene therapy usually rely on the use of transcranial injections, resulting in invasive damage and limited spatial coverage 5. Developing a non-invasive technique for perturbing neuronal activity with high spatial resolution has been a long-standing challenge in brain disease even with psychiatric disease treatments.

Ultrasound (US) has arisen as an alternative method that can overcome the trade-offs faced by conventional neuromodulation strategies; it can effortlessly enable penetration of an intact skull to modulate targeted brain regions, including the visual cortex, somatosensory cortex, motor cortex, and even the thalamus 6-9. However, it is difficult to predict the effect of US on neuronal activity because of the sub-millimeter spatial resolution of ultrasound, which may result in concurrent simulation of different types of neurons. To improve on the inaccuracy of US in neuromodulation, lbsen *et al.* used gas-filled microbubbles (MBs) with US for targeted stimulation of the neuronal cells in C. *elegans* that expressed the TRP-4 mechanotransduction channel 10. They successfully manipulated the function of sensory neurons and identified a role for unidentified PVD sensory neurons. However, the short lifespan of MBs *in vivo* (< 10 min) and the difficulty in transporting MBs into extravascular tissues have limited its application. Recently, several groups have attempted implanting mechanosensitive ion channels, including Piezo1 and Mscl, into an *in vitr*o cell culture system to improve the US-sensing ability of specific cells 11, 12. However, the US frequencies utilized in these studies (30 MHz and 43 MHz) limit the applicability *in vivo* due to the high attenuation. Until now, there has not been a feasible sonogenetic system using low-frequency and low-pressure US to remotely control genetically modified mammalian tissue.

To circumvent the obstacles of current sonogenetic systems, we recently established a sonogenetic approach for controlling cellular activities using medical US excitation 13. Our strategy uses naturally occurring US-responsive proteins. We focused on a transmembrane protein, Prestin. Previous studies had reported that Prestin resides in the outer hair cells (OHCs) of mammalian cochlea and drives the electromotility of OHCs, which seems to be important for high-frequency hearing 14-16. Interestingly, heterologous expression of Prestin in mammalian cell lines results in several hallmarks of OHCs, suggesting that Prestin may act as an electromechanical transducer *per se*. Under the assumption that several parallel amino acid substitutions of Prestin may be involved in adaptive US hearing 17-22, we analyzed the Prestin protein sequence among 5 non-echolocating and 10 echolocating species and found that the 7^th^ and 308^th^ amino acids often switch from N to T and N to S, respectively. To test whether these evolutional amino acid substitutions play any roles in adaptive US sensing, we introduced two evolution-based mutants, N7T and N308S, into the Prestin protein of non-echolocating mice, including Prestin mutants containing a single substitution, Prestin (N7T) and Prestin (N308S); and a mutant containing two substitutions, Prestin (N7T, N308S). Our data demonstrated that expression of murine Prestin (N7T, N308S) in mammalian cells produced ~11-fold better US sensitivity compared to control cells under low-frequency (0.5 MHz), low-energy (0.5 MPa and 0.1% of duty cycle), and transient (3 s) US conditions 13. However, the biggest limitation of this sonogenetic system is that gene delivery of Prestin (N7T, N308S) into mouse brain still depends on injection with the adeno-associated virus system 13.

**Figure 1:**
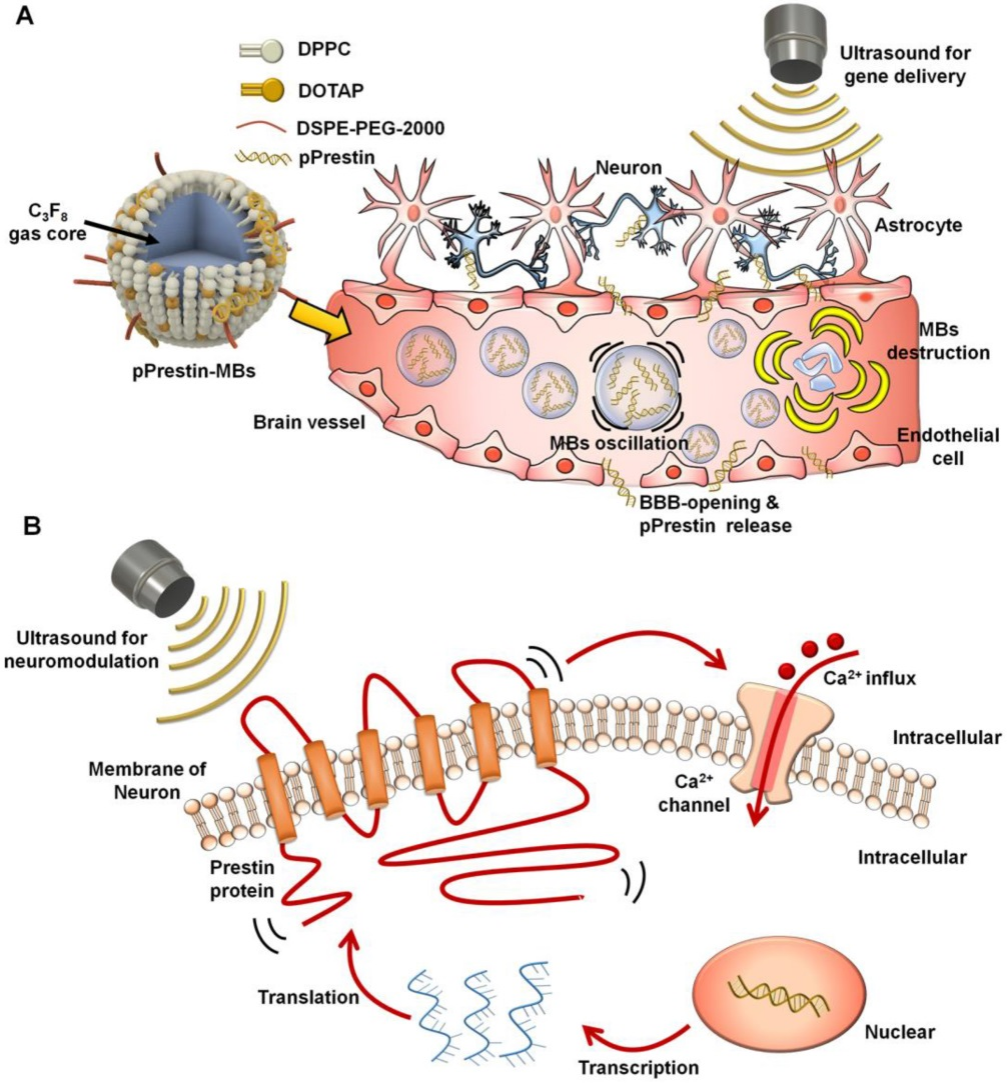
Illustration paradigm of this study. (A) An engineered ultrasound -responsive DNA (Prestin plasmid, pPrestin) was transcranially transfected by ultrasound with pPrestin-MBs. (B) Transcranial activation of the Prestin-expressing neurons by ultrasound.

The ultra-high US sensitivity of mutant Prestin (N7T, N308S) enables transcranial targeting of neurons buried in deep brain regions by low-frequency ultrasound. To take advantage of this, we combined three newly established approaches: US-induced blood-brain barrier (BBB) disruption for spatial targeting 23-27, microbubble (MBs) gene carriers for transporting genes to a specific area 28, 29, and an engineered US-responsive protein (mutant Prestin (N7T, N308S)) for stimulation of targeted neurons by US. In this paradigm, the experimental processes include three stages: (1) design of US-responsive DNA (Prestin plasmid, pPrestin)-loaded MBs (pPrestin-MBs); (2) non-invasive transfection of pPrestin into the brain of mice with US (Fig. 1A); and (3) activation of the transfected neurons by low-frequency US (Fig. 1B). Future work will include application of this technique to treat other neurodegenerative disorders, or as a new tool for exploring brain neuroscience.

## Method

### Plasmid preparation

Expression vectors for pPrestin with green fluorescence protein (Venus) driven by the cytomegalovirus promoter were prepared as described in our previous study 13. All plasmid DNA was purified using the Plasmid Maxi Kit (NucleoBond Xtra Maxi EF, Macherey-Nagel, Düiren, Germany). The purity of plasmid DNA was verified by ensuring the A260/A280 ratio was between 1.8 and 1.9 for use in the following experiments by spectrophotometer (NanoDrop 2000, Thermo Fisher Scientific, IL, USA).

### Preparation of pPrestin-loaded microbubbles (pPrestin-MBs)

The MBs were fabricated by dissolving the dipalmitoyl-phosphatidyl-choline (DPPC) (Avanti Polar Lipids, AL, USA), 1,2-distearoyl-sn-glycero-3-phosphoethanolamine-N-[methoxy(poly(ethyleneglycol))-2000] (DSPE-PEG2000) (Avanti Polar Lipids) and 1,2-dipalmitoyl-3-trimethylammonium-propane (DPTAP) (Avanti Polar Lipids) in chloroform at a molar ratio of 9:2:1, and then draining to generate a lipid film 30. The film was then dissolved with 1 wt% glycerol-containing phosphate-buffered saline (PBS). Subsequently, the solution was degassed and refilled with perfluoropropane (C_3_F_8_). The MBs were formed by intense mechanical shaking using an agitator for 45 s. Finally, to separate from unreacted lipids, centrifugation was applied to the MBs at 6,000 rpm (2000 g) for 3 min.

Plasmid DNA (0-10000 ng) was gently mixed with 10^8^ MBs for 30 min, and was centrifuged at 6,000 rpm (2000 g) for 1 min for separating unattached plasmid DNA. The normal MBs without plasmid DNA payload were prepared for comparison.

### Properties of the pPrestin-MBs

#### Concentration, size distribution, and payload

The concentration and size distribution of the pPrestin-MBs were measured by a Coulter counter (Multisizer 3, Beckman Coulter, FL, USA). The structure of the pPrestin-MBs was detected by a microscope (Eclipse Ti, Nikon, Tokyo, Japan). To visualize the pPrestin loaded onto the shell of MBs, pPrestin was labeled with red fluorescence dye (Rhodamine, Label IT Rhodamine kits, Mirus, WI, USA) and used to prepare pPrestin-MBs. The plasmid DNA payload of pPrestin-MBs was quantified by spectrophotometer.

#### Acoustic properties

**Figure 2:**
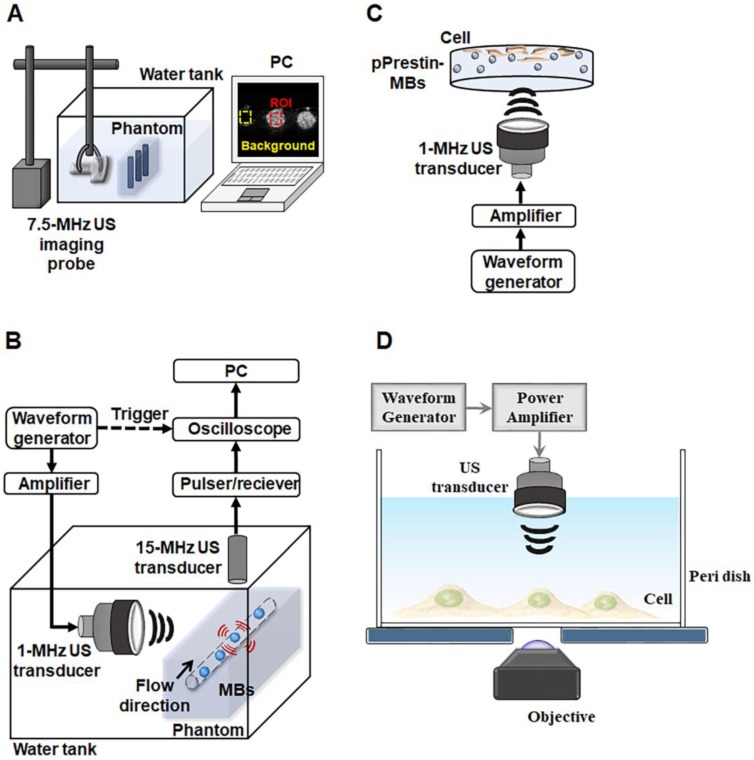
Experimental setup. (A) Acoustic stability evaluated by sonographic B-mode imaging. (B) Acoustic destruction threshold detected via passive cavitation detection. (C) Cellular gene transduction by pPrestin-MBs with 1-MHz US. (D) 0.5-MHz US-stimulated pPrestin-transfected cells and recoding the calcium influx of those cells concurrently via live-cell microscopic imaging.

The acoustic stability of the pPrestin-MBs was evaluated by measuring the echogenicity from B-mode images. The pPrestin-MBs were loaded into a 2% agarose phantom and imaged by a 7.5-MHz sonographic system (model t3000, Terason, MA, USA, Fig. 2A) at 37 °C for 1 h (imaging interval: 5 min). The echogenicity of the pPrestin-MBs, unloaded MBs, and saline were determined from the acquired B-mode images by MATLAB^TM^ software (The MathWorks, Natick, MA, USA).

The acoustic destruction threshold of pPrestin-MBs was assessed using a passive cavitation detection method (Fig. 2B). The pPrestin-MBs solution was injected into the agarose phantom with tunnel (diameter: 200 µm) by a syringe pump (KDS120, KD Scientific, New Hope, PA, USA) at 40 mm/s. Then, the pPrestin-MBs were sonicated by a 1-MHz focused US transducer (model V303, Olympus, MA, USA, cycle number: 5000, pulse repetition frequency: 1 Hz, acoustic pressure: 0-1.1 MPa) and the broadband signal was received by a 15-MHz focused US transducer (V315, Olympus). The 1-MHz focused US transducer was triggered by an RF power amplifier (Model 150A100B, Amplifier Research, Hazerswoude-Dorp, Netherlands) and a waveform generator (AFG3251, Tektronix, OR, USA). The broadband signal was processed by Fourier transform and the signal between 10 MHz and 20 MHz was integrated to evaluate the activity of the inertial cavitation. The acoustic pressures utilized in this study were calibrated by a polyvinylidene difluoride type hydrophone (model HGL-0085, ONDA, CA, USA; calibration range = 1-40 MHz) in a water tank filled with degassed and distilled water at 25 °C.

### *In vitro* experiment

#### pPrestin transfection

SH-SY5Y cells were cultured in Dulbecco's modified Eagle's medium containing growth factor F12 (DMEM/F12) (Gibco, NY, USA) supplemented with 10% Fetal Bovine Serum (FBS, Gibco), 1% penicillinestreptomycin (Gibco) and 1.2 g/L NaHCO_3_ at 37 ℃. One day before experiment, a total of 3✕10^4^ cells were seeded in a 24-well plate in 500 µL medium (DMEM with 10% FBS). For the experiment, a pPrestin-MBs (concentration: 2✕10^8^ MBs/mL; pPrestin: 7500 ng) solution was added to the medium. The cell dish was flipped upside down and was sonicated by 1-MHz focused US (cycle number: 50-10000, pulse repetition frequency: 0.5-5 Hz, duration: 1 min, acoustic pressure: 0.1-0.7 MPa) to facilitate the pPrestin transfection (Fig. 2C). Four hours after 1-MHz focused US exposure, cells were washed with DPBS (Dulbecco's Phosphate-Buffered Saline, Biological Industries, CT, USA) and cultured in fresh medium. The gene transfection rate was assessed by counting the number of green fluorescence (Venus) positive cells at 48 h after gene transfection by flow cytometry (FACScalibur, BD Biosciences, CA, USA). Cell viability was determined using the Alamar Blue indicator (AbDSerotec, Oxford, UK).

#### Ultrasound-stimulated pPrestin-transfected cells

Previous studies had shown US with nanoparticles could evoke calcium influx in SH-SY5Y cells 31, 32. The calcium influx of cells was used as a readout in response to the mechanical stimulation of ultrasound wave. To record the calcium influx of cell, the calcium biosensor cyan fluorescence protein (CFP)-R-GECO was co-transfected into the cells with a commercialized transfection reagent (TransIT®-LT1, Mirus). Forty-eight hours post-transfection with 1-MHz focused US and pPrestin-MBs, the cell co-expressing CFP-R-GECO and Prestin were sonicated by 0.5-MHz focused US (cycle number: 2000, pulse repetition frequency: 10 Hz, duration: 3 s, acoustic pressure: 0.5 MPa; V389, Olympus). In order to record the calcium influx of cell in real-time, the 0.5-MHz focused US transducer was confocally positioned with the objective of the microscope (Fig. 2D). The images were acquired starting from 10 s before 0.5-MHz focused US sonication, and lasting for a total of 70 s. Cells without pPrestin transfection were used as the control group.

The acquired fluorescence images were analyzed with MATLAB^TM^ software. Each cell was selected and analyzed for fluorescence intensity during the time lapses; subsequently, the readings were converted in ΔF/F_0,_ and plotted on the ΔF/F_0_ graphs. F_0_ was denoted as the fluorescence intensity before US stimulation, and ΔF was denoted as the fluorescence intensity after US stimulation subtracted from F_0_. The experiments were carried out in triplicate (at least the response of 40 cells for all the conditions was analyzed).

### *In vivo* study

#### Animal preparation

All animal procedures were following the guidelines of the National Tsing-Hua University animal committee (IACUC approval number: NTHU107034). Healthy C57BL/6J mice (20-30 g, National Laboratory Animal Center, Taipei, Taiwan) were employed to evaluate the degree and safety of BBB opening, gene transfection, and neuromodulation. Before experiments, a mixture of Rompun 2% (Bayer HealthCare, LeverKusen, Germany) and Zoletil 50 (Virbac, Carros, France) was injected intraperitoneally to anesthetize the animals.

#### Confirmation of BBB-opening by ultrasound and pPrestin-MBs

**Figure 3:**
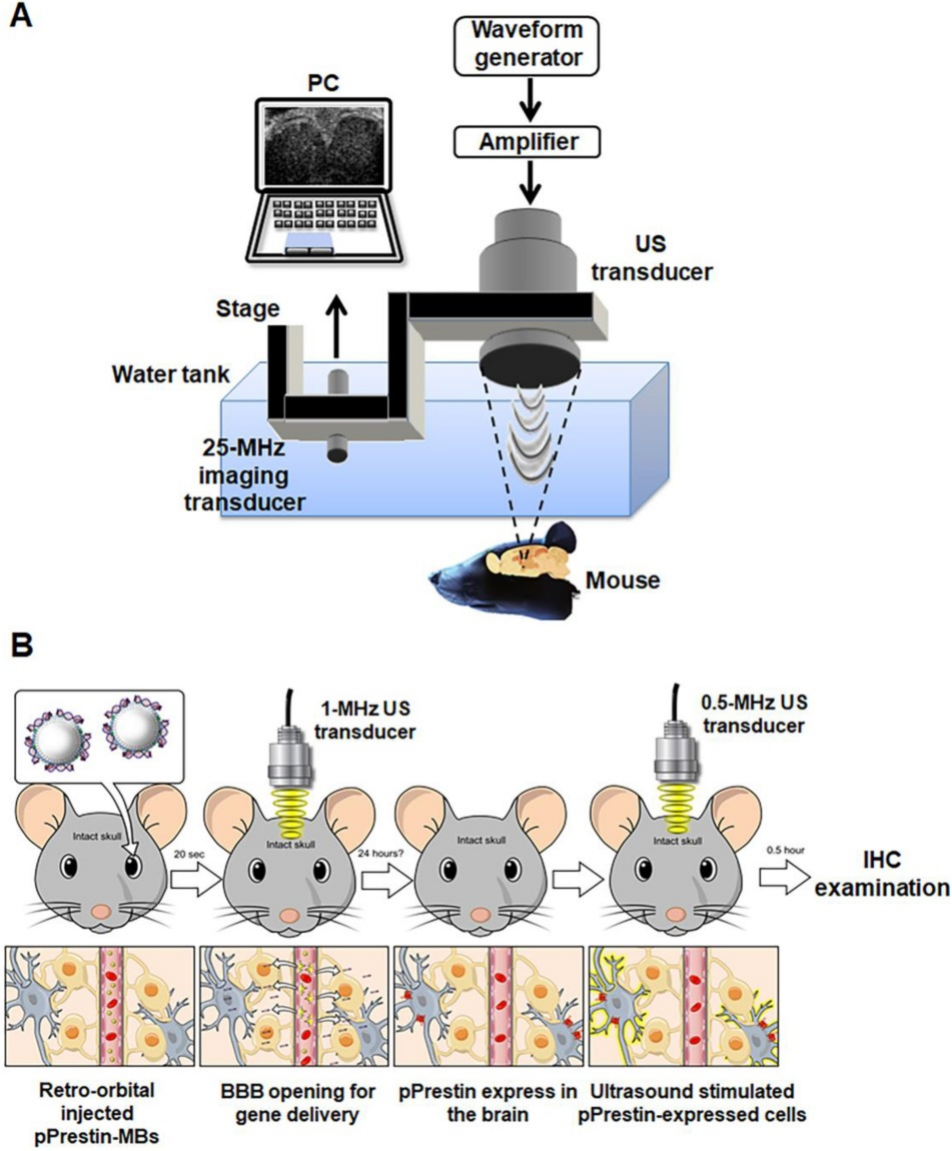
(A) *In vivo* experimental setup and (B) flowchart for the animal study.

An Evans blue (EB) dye (20 µL, 75 mg/kg, Sigma-Aldrich, MO, USA) solution was retro-orbitally injected and circulated for 10 min. MBs solution (100 µL, 1✕10^8^ MB/mL, pPrestin: 7500 ng) was administrated by retro-orbital injection. Twenty seconds later, the 1-MHz focused US (cycle number: 5000, pulse repetition frequency: 1 Hz, duration: 1 min, acoustic pressure: 0.3-0.7 MPa) was guided by 25-MHz US imaging system and transcranially delivered to the left brain of mice (n=12) (Fig. 3A) 33. After 30 min of circulation for EB extravasation, the brains were removed from the animals and sliced into coronal sections. Hematoxylin and eosin (H&E) and terminal deoxynucleotidyl transferase biotin-dUTP nick end labeling (TUNEL, ApopTag kit, Intergen Co., Purchase, NY, USA) staining were employed to verify concerns such as hemorrhage and apoptotic cells, respectively.

#### pPrestin transfection *in vivo*

Figure 3B illustrates the flowchart used for the animal transfection experiment. Animals were sacrificed following 1-MHz focused US and completion of pPrestin-MB treatment (1, 2, 7, 14, and 21 days; 60 s, 120 s, and 240 s; n=4 in each condition) and were perfused with 4% paraformaldehyde. The frozen brains were sliced into coronal sections. The activity and location of gene transfection were identified using the intracerebral expression of green fluorescence protein (Venus) via microscope. The cellular nuclei were labelled by DAPI (GTX30920, GeneTex, Inc., TX, USA). The contralateral brain without gene transfection was used for comparison. The expression of Prestin was estimated by calculating the intensity of green fluorescence within ROI.

#### Ultrasound-stimulated pPrestin-transfected cells

The pPrestin transfected mice (n=6) were stimulated by a 0.5-MHz focused US transducer at 48 h after gene transfection. The setup was the same as that used for gene transfection. 0.5-MHz focused US (cycle number: 5000, pulse repetition frequency: 1 Hz, duration: 1 min, acoustic pressure: 0.5 MPa) was guided by the 25-MHz US imaging and transcranially delivered to the brain of mice (Fig. 3A). Two different experiments were performed: (1) Examining if Prestin expression would enhance the US sensibility. Both hemisphere brains (left: Prestin-expression; right: non Prestin-expression) of animal underwent US excitation (n=4). The signal intensity of c-Fos (tagged with red fluorescence, Dylight 594) in the Prestin expression area and contralateral area after US excitation were evaluated.; (2) Examining if US excitation only could activate Prestin-expressing cells. US was only delivered in the Prestin-expressing hemisphere of the brain, with the contralateral brain used as a comparison (n=4). The overlap between Prestin-expressing cells (green fluorescence signal, Venus) and c-Fos signals (red fluorescence signal, Dylight 594) with or without US excitation (n=6) were estimated by calculating the number of Venus positive cells and Venus/c-Fos double positive cells in different animal groups.

#### Immunohistochemistry staining (IHC)

The successful stimulation of pPrestin-transfected cells was verified by c-Fos IHC staining 34. After 0.5-MHz FUS stimulation, the animals were sacrificed and perfused with 4% paraformaldehyde. The brains of mice were removed and sliced into 15-μm sections. The sections were then incubated in primary rabbit anti-c-Fos antibody (1:1000; SYSY, Goettingen, Germany) diluent overnight. Subsequently, the sections were incubated for 1 h in Dylight 594 conjugated anti-rabbit secondary antibody (1:200, GeneTex, Inc.) diluent followed by several washes in PBS. The cellular nuclei were labelled by DAPI. Finally, coverslips were added to the slides with fluorescent mounting medium and stored flat in the dark at -20ºC.

### Statistics

All results are denoted as the mean ± standard deviation. All statistical evaluations were carried out with unpaired two-tailed Student's t-tests. A p-value of less than 0.05 was accepted as representing a statistically significant difference.

## Results

### Properties and DNA loading capability of pPrestin-MBs

**Figure 4:**
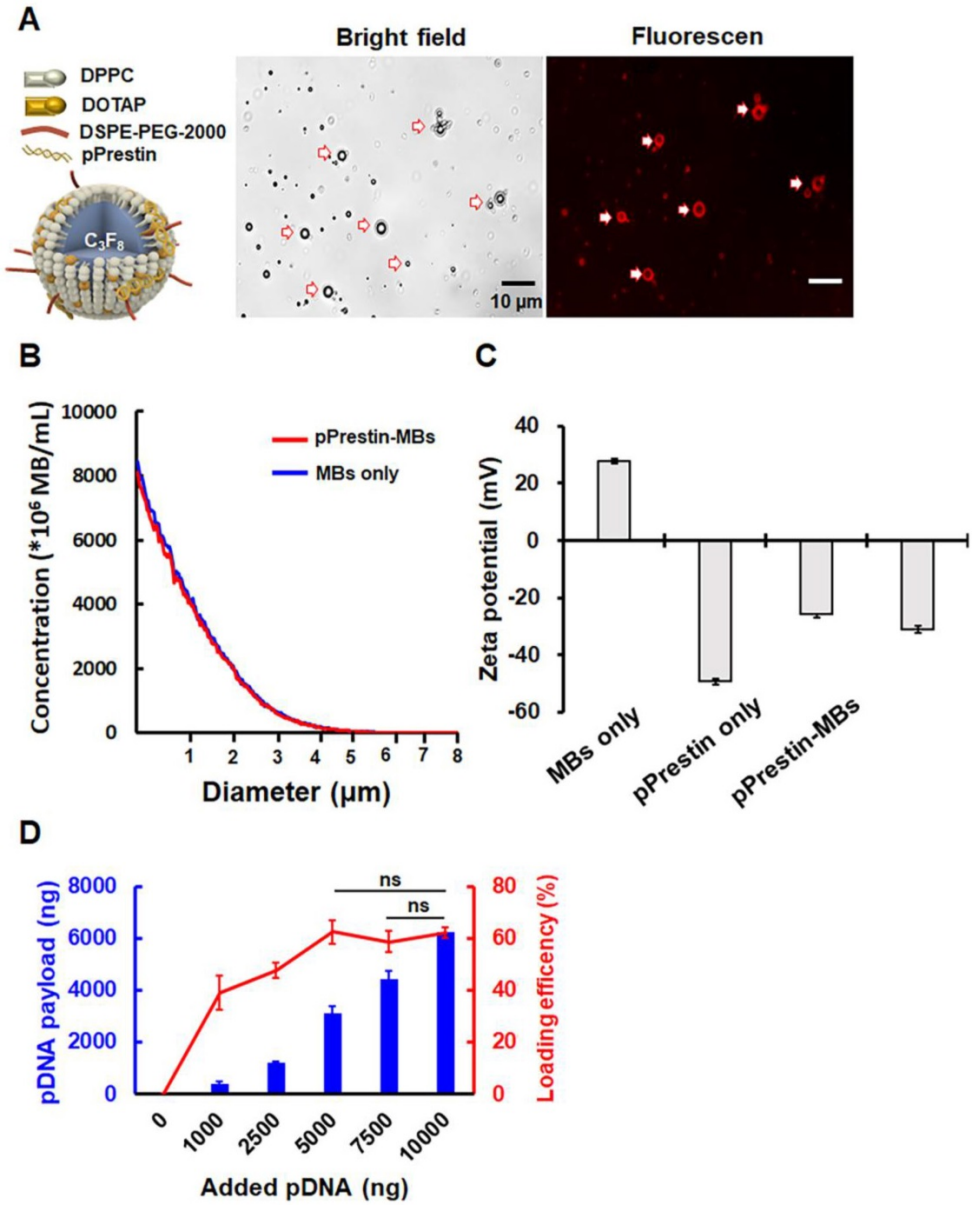
(A) Left: Illustration of the structure of pPrestin-MBs; right: microscopic images of pPrestin-MBs. (B) Size and concentration of pPrestin-MBs. (C) Zeta potential of pPrestin-MBs. (D) DNA payload and efficiency of pPrestin-MBs. Data are shown as the mean ± standard deviation for 4 independent experiments.

Figure 4A illustrates the structure of pPrestin-MBs. The co-localization of spherical DNA (tagged by Rhodamine dye) signals and MB morphology suggested successful loading of DNA onto MBs (Fig. 4A). The mean diameter and mean concentration of unloaded MBs were 1.1±0.1 μm and (17.4±0.7) × 10^9^ MB/mL, respectively (Fig. 4B). When loaded with pPrestin, the mean diameter and mean concentration of pPrestin-MBs were 1.1±0.2 μm and (16.0±0.2) × 10^9^ MB/mL, respectively. The dramatic decrease in zeta-potential of MBs from positive to negative after DNA loading (+27.9±0.8 mV to -35.1±1.6 mV) also confirmed the DNA coating of the MB shell (Fig. 4C). The DNA payload efficiency was measured by calculating the ratio of bound DNA on a bulk number of MBs (10^8^) at different DNA amounts. When mixing 1000-5000 ng DNA with MBs, there was an increase in DNA loading efficiency from 40.9±2.4% to 50.8±1.3%. When mixing more than 7500 μg of DNA to the MBs, the DNA loading efficiency plateaued at 61.6% (Fig. 4D). However, the amount of DNA bound to MBs can still be improved by increasing the amount of added DNA.

### Acoustic properties of pPrestin-MBs

**Figure 5:**
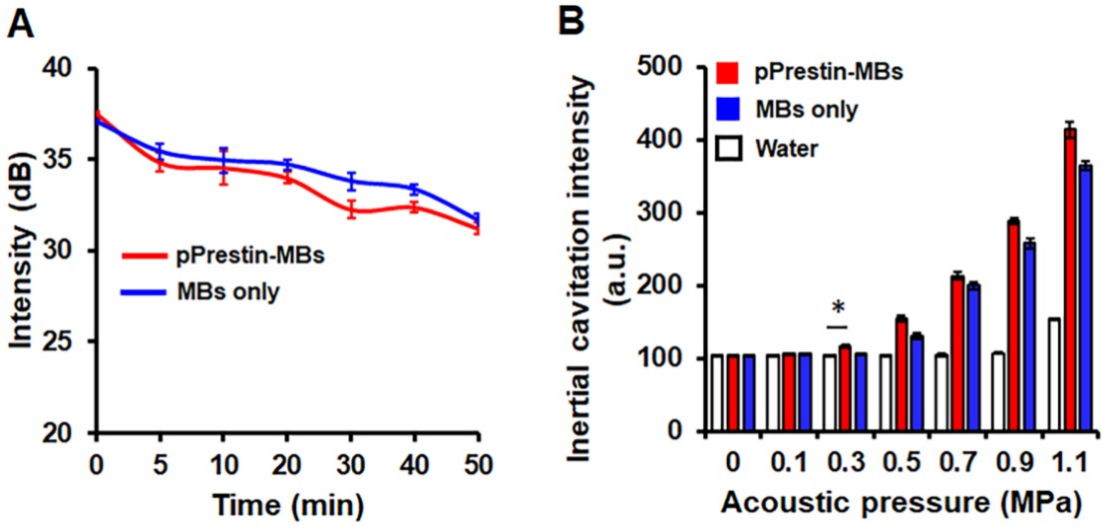
(A) Acoustic stability of pPrestin-MBs and unloaded MBs. (B) Acoustic destruction threshold of pPrestin-MBs and unloaded MBs. *: *p*<0.05. Data are shown as the mean ± standard deviation for 4 independent experiments.

The existence of a gaseous core within MBs plays a key role in US-triggered cargo release. We thus applied sonographic imaging to verify the stability of pPrestin-MBs since the B-mode imaging contrast of MBs is dominated by the gaseous core within MBs. The signal intensity of pPrestin-MBs gradually decreased with time under 37 ºC (0 min: 37.1±0.1 dB to 50 min: 31.6±0.4 dB) (Fig. 5A), and MBs without DNA loading also showed the same decrease (0 min: 37.5±0.1 dB to 50 min: 31.2± 0.3 dB), suggesting natural gas diffusion from MBs. Since gene delivery from pPrestin-MBs needs the destruction of pPrestin**-**MBs by US, the next issue was to evaluate the acoustic destruction threshold of pPrestin**-**MBs. The MBs emit acoustic broadband signals when disrupted by ultrasound (referred to as inertial cavitation). Thus, a 15-MHz US transducer was selected to acquire the instantaneous acoustic signals when pPrestin-MBs were sonicated by 1-MHz US. The inertial cavitation of pPrestin-MBs appeared from 0.3 MPa, demonstrating the destruction of pPrestin-MBs. (Fig. 5B).

### *In vitro* gene transfection efficiency and cell viability

**Figure 6:**
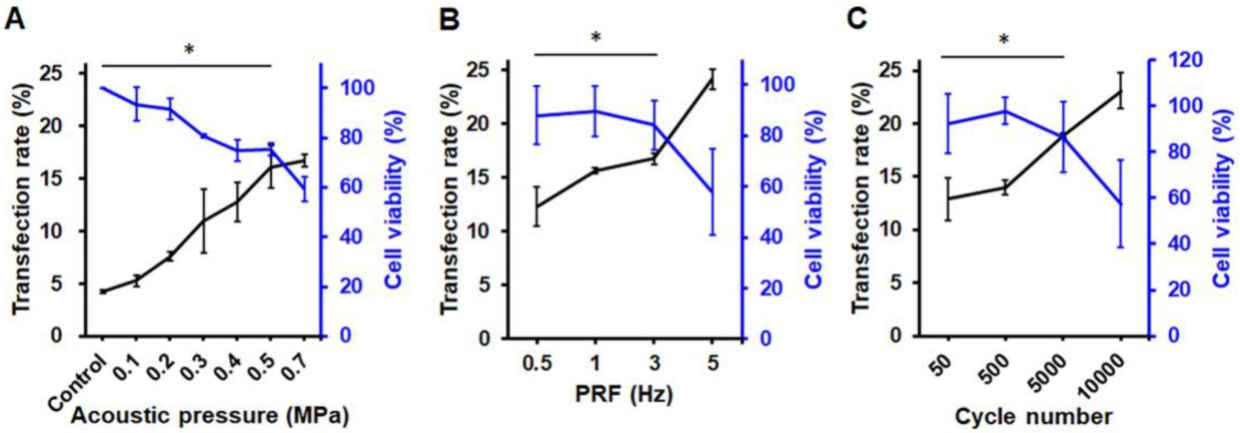
Transfection rate (measured by flow cytometry) and cell viability with different 1-MHz US parameters. (A) Acoustic pressure. The maximum green fluorescence protein expression occurred at acoustic pressure of 0.5-0.7 MPa. (B) PRF. The PRF threshold of gene delivery was 0.5 Hz, and it peaked at 5 Hz. (C) Cycle number. The maximum transfection rate occurred at 10000 of cycle number. *: *p*<0.05. Data are shown as the mean ± standard deviation for 6 independent experiments.

The pPrestin transfection capabilities of the proposed gene delivery system were quantified by flow cytometry. We found no Prestin expression following US exposure with 0-0.2 MPa (< 7%) (Fig. 6A). Increasing the pressure from 0.2-0.5 MPa produced a profound increase in the transfection rate (0.3 MPa: 10.9±2.9%; 0.4 MPa: 12.8± 1.8%; 0.5 MPa: 16.1±2.0%). This indicates that an acoustic pressure greater than 0.3 MPa is required for gene transfection due to inertial cavitation of pPrestin-MBs that starts at 0.3 MPa (Fig. 6A). However, increasing the acoustic pressure from 0.5 MPa to 0.7 MPa produced a slightly increased transfection rate (16.7±0.6%), but decreased cell viability (75.2±0.1% to 59.3±0.1%).

The effect of PRF was then investigated by maintaining an acoustic pressure at 0.5 MPa. The results suggest that sonicating ultrasound with 0.5 Hz of PRF only achieves slight pPrestin transfection (12.3±1.8%) (Fig. 6B). Increasing PRF (1 Hz and 3 Hz) resulted in a minor improvement in transfection rate (15.6±0.2% to 16.7±0.5%), while maintaining high cell viability (89.7±0.1%, and 84.1±0.1%). A significant increase in transfection rate appeared at 5 Hz of PRF (24.2±0.9%), but there was a drop in cell viability (57.8±0.2%).

Sonicating US with 50 cycles could achieve Prestin transfection (12.9±2.1%) (Fig. 6C). Increasing the cycle number from 500 to 5000 increased the transfection rate (14.0±0.1% to 18.9±0.2%). The increase in pulse length also caused a significant decrease in cell viability (97.9±0.1% to 86.4±0.2%). Although the transfection rate could be up to 23.1±1.7% with 10000 cycles, the increase in pulse length also caused a significant decrease in cell viability (50.7±0.2%). Our results show that transfection rate is strongly dependent on acoustic pressure, with the optimal acoustic parameters for gene transfection being 0.5 MPa, 5 Hz of PRF, and 5000 for cycle number.

### Ultrasound-driven pPrestin-expressing cells induce Ca^2+^ Transients

**Figure 7:**
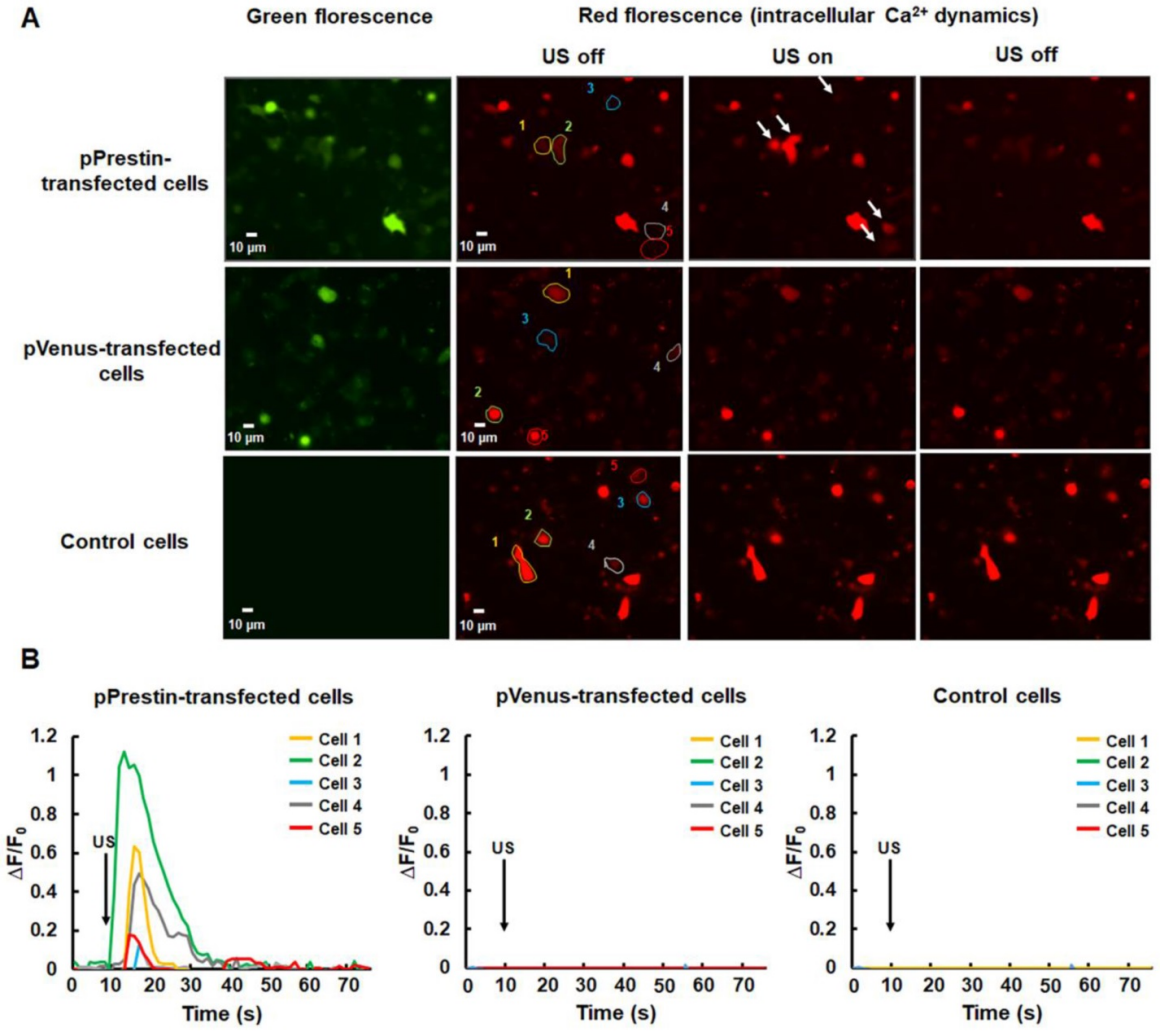
(A) Calcium imaging of SH-SY5Y cells with or without pPrestin expression in response to 0.5-MHz US stimulation (movie S1). (B) Time course of the ΔF/F_0_ traces. Arrows indicate the initiation of the 0.5-MHz US pulse (at t = 10). Data was acquired from 6 independent experiments.

After the evaluation of the pPrestin transfection into cells, we monitored the intracellular Ca^2+^ dynamics in response to 0.5-MHz US stimulation. The control group consisted of cells that were not transfected with pPrestin. The data demonstrated that expression of pPrestin did not induce spontaneous calcium responses in the absence of US stimulation (Fig. S1). We observed that administration of 0.5-MHz US resulted in a cytoplasmic Ca^2+^ influx in pPrestin-expressing cells from the extracellular space (Fig. 7A, Fig. S2) (∆F/F_0_ = 0.7±0.1%, n =5), but not in the pVenus-expressing cells (∆F/F_0_ = 0.1±0.1%, n = 5) and control group (∆F/F_0_ = 0.1± 0.1%, n = 5) (Fig. 7B, movie S1). Moreover, the cell viability was not affected by US stimulation (Fig. S3). This result indicates that the US-sensing ability of pPrestin would be not affected by our proposed gene delivery platform.

### *In vivo* gene transfection

#### Local BBB-opening capability by ultrasound with pPrestin-MBs

**Figure 8:**
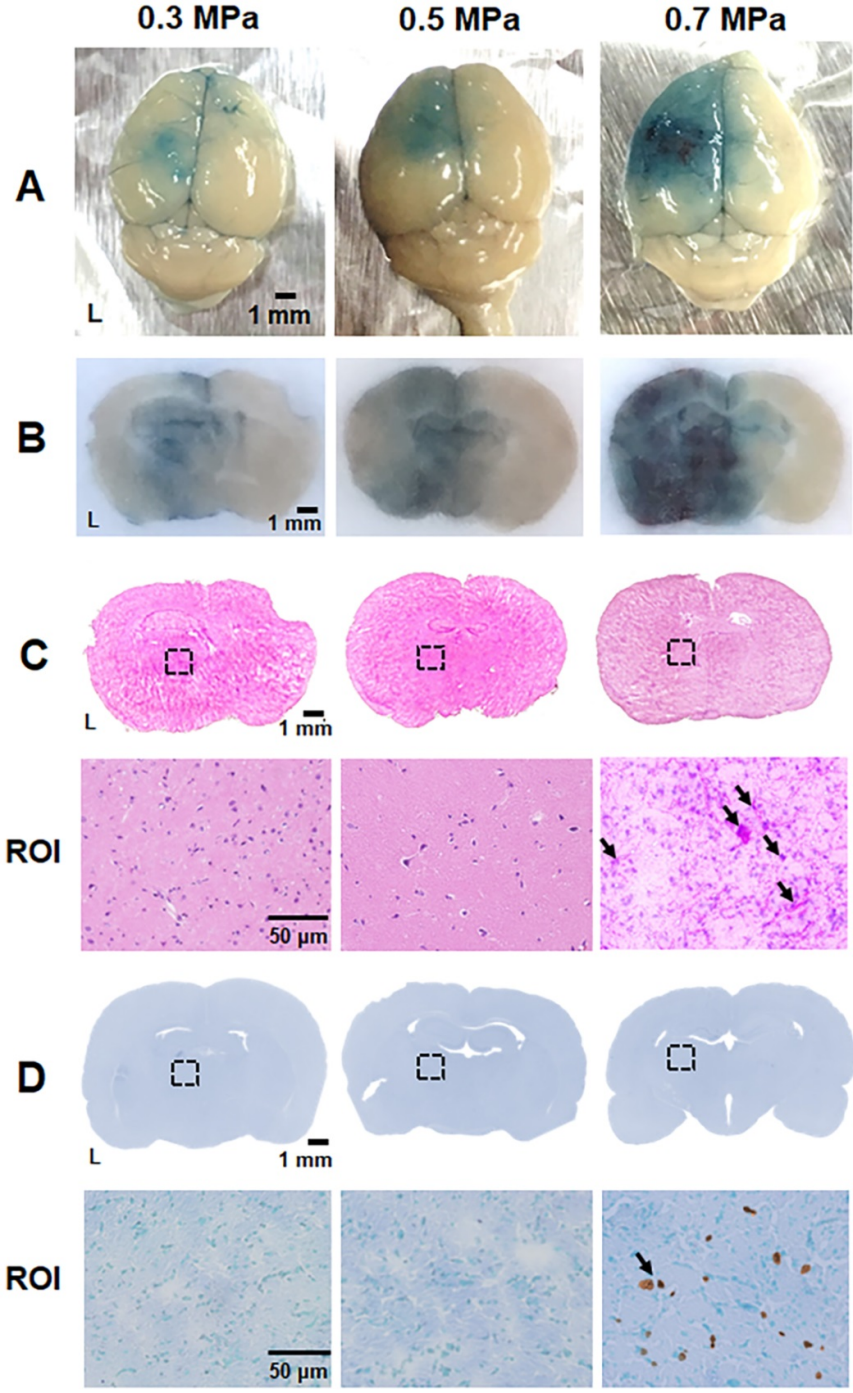
Parameter optimization for BBB disruption. (A) Brain surface and (B) histologic section for estimate the degree of BBB disruption level by EB extravasation (blue area). (C) Top: the corresponding H&E staining from (B) was employed to assess brain damage; bottom: magnification (200×) of BBB-disruption area from ROI (black dot rectangle) to visualize the erythrocyte extravasation (black arrow). (D) Top: the corresponding TUNEL staining from (B) was employed to assess brain damage; bottom: magnification (200×) of BBB-disruption area from ROI to detect the apoptosis cells (black arrow). n = 4 per group.

The optimal acoustic parameters for gene transfection were determined using *in vitro* experiments, and we explored the safety issues and feasibility of *in vivo* gene transfection with these parameters in a healthy mouse model. The BBB within the cerebrovascular is the major hurdle in brain gene delivery. Therefore, the extent of BBB disruption via US with pPrestin-MBs was verified using EB dye. EB dye leaks from BBB-disrupted vessels allowing the level and area of BBB-disruption to be estimated visually. Figure 8A-C demonstrates that minor and safe BBB-disruption occurs after US sonication with 0.3-0.5 MPa, as confirmed by H&E staining. Both the region and level of BBB-disruption was additionally boosted at 0.7 MPa, but was accompanied by severe erythrocyte extravasation and cellular apoptosis in the treated area due to intense inertial cavitation of MBs (Fig. 8C-D). To achieve maximum efficiency of gene delivery with high safety, an acoustic pressure of 0.5 MPa was chosen for subsequent *in vivo* gene transfection experiments.

#### *In vivo* gene transfection ability

**Figure 9:**
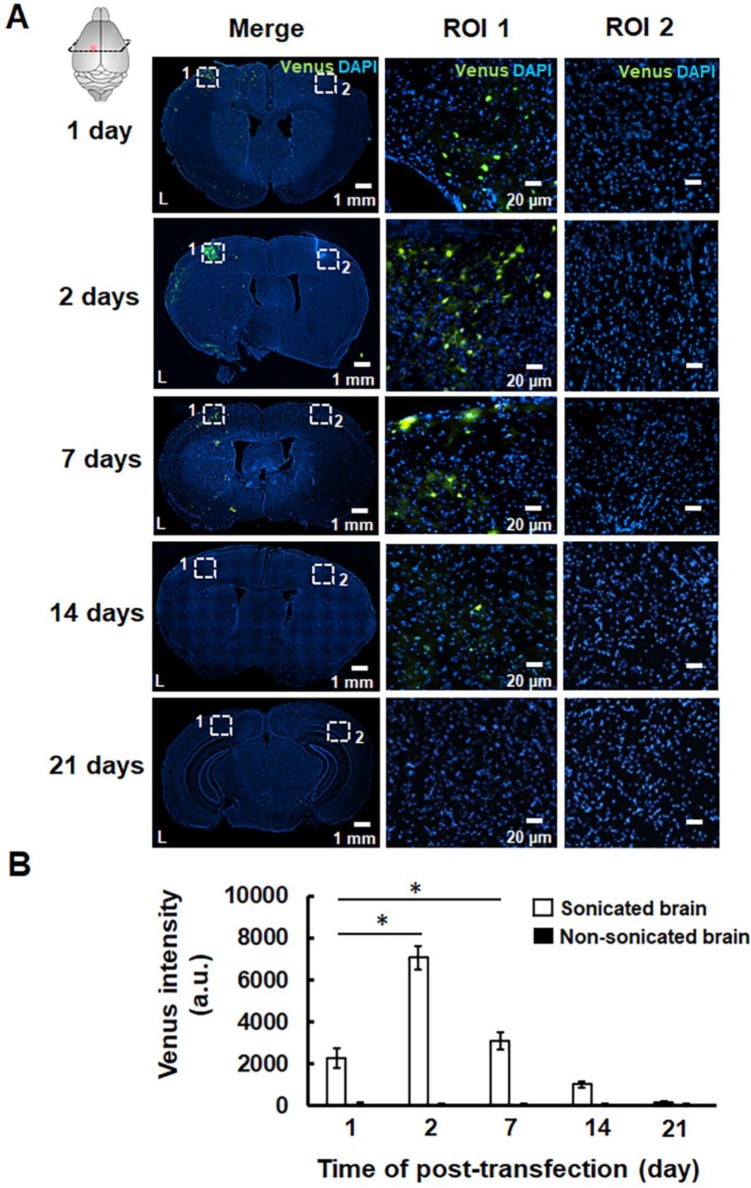
(A) pPrestin-MBs with 1-MHz US (0.5 MPa) were used for gene delivery to the mouse brain at 1, 2, 7, 14, and 21 days post-transfection. Successful gene transfection was verified by the expression of green fluorescence protein (Venus). Left: whole brain section; middle: magnification (200×) of sonicated site from ROI 1 (white dot rectangle); right: magnification (200×) of contralateral non-sonicated site from ROI 2. (B) Time course of gene expression activities expressed as intensity of green fluorescence protein in the region of interest. *: *p*<0.05. Data are shown as the mean ± standard deviation for 5-9 different sections (n = 4 per group).

**Figure 10:**
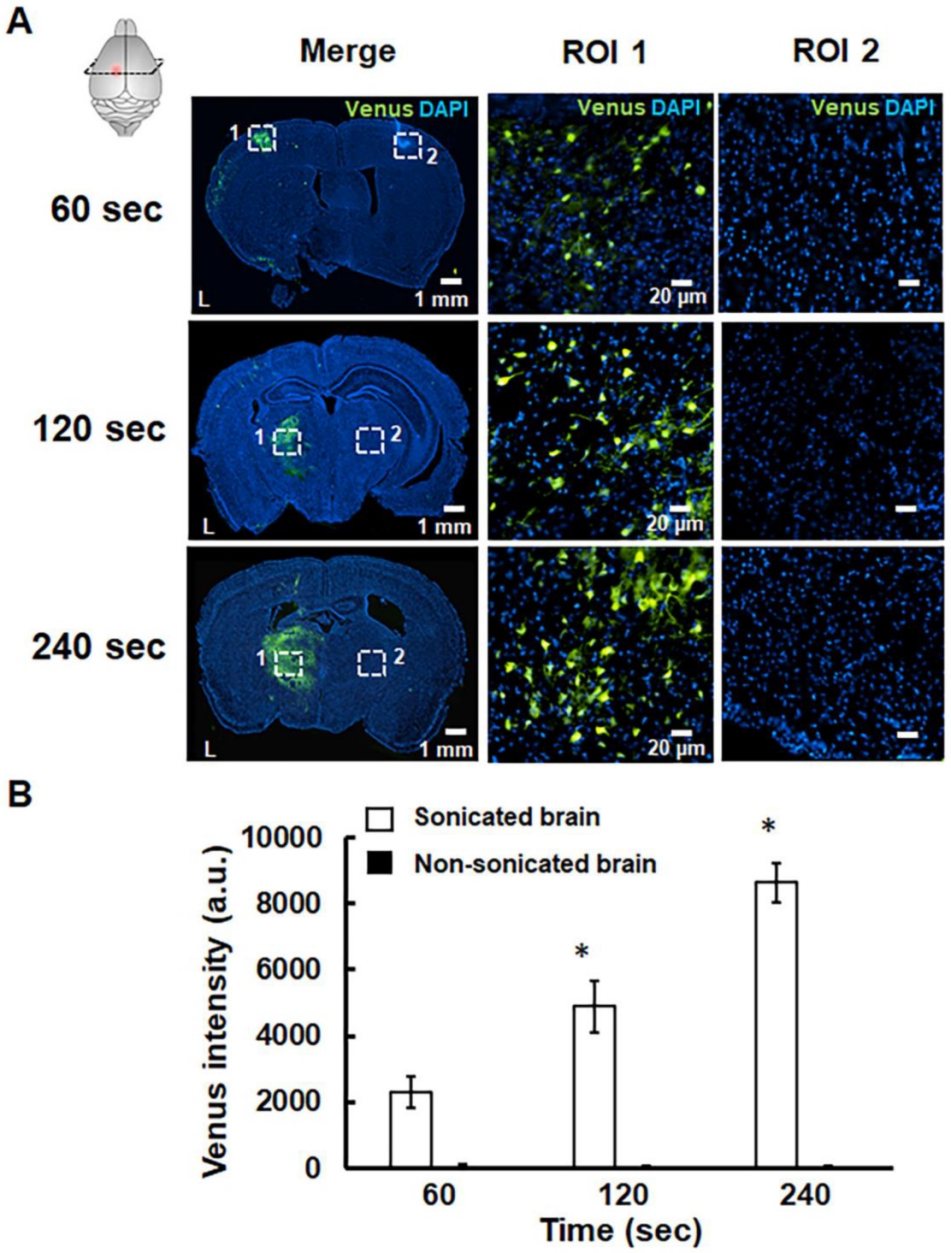
(A) pPrestin-MBs with 1-MHz US (0.5 MPa) was used for gene delivery to the mouse brain at 2 days posttransfection with sonication times of 60 s, 120 s, and 240 s. Successful gene transfection was verified by expression of green fluorescence protein (Venus). Left: whole brain section; middle: magnification (200×) of sonicated site from ROI 1 (white dot rectangle); right: magnification (200×) of contralateral non-sonicated site from ROI 2. (B) Gene expression activities at different sonication times. Measurements of the gene expression are expressed as intensity of green fluorescence protein in the region of interest. *: *p*<0.05. Data are shown as the mean ± standard deviation for 5~9 different sections (n = 4 per group).

The successful pPrestin transfection was evaluated using the intracerebral expression of green fluorescence protein (Venus) by microscopic image. The effect of transfection time and sonication number for brain gene transfection was assessed in the following section. The brains of treated animals were removed and sliced at different time points (1, 2, 7, 14, and 21 days). Figure 9 indicates that green fluorescence signals start to appear at 1 day after treatment (2300.5±465.9 a.u.). The distribution of green fluorescence signals peaked 2 days following gene transfection and then decreased with time (2 days: 7071.8±554.6 a.u.; 7 days: 3099.8±432.6 a.u.; 14 days: 1019.6±149.7 a.u.; 21 days: 189.5±57.9 a.u.). In contrast, no green fluorescence signal was detected in the non-sonicated brain (1 day: 113.3±36.2 a.u.; 2 days: 96.5±19.1 a.u.; 7 days: 83.3±28.0 a.u.; 14 days: 77.0±16.1 a.u.; 21 days: 86.2± 10.1 a.u.). The pPrestin-expressing cells included vascular endothelial cells (20.2±4.6%), neuron cells (68.3±6.9%), glutamatergic neurons (26.1±5.8%), dopaminergic neurons (16.5±10.8%), and astrocyte (2.1±0.4) (Fig. S4). After examining the time point for maximizing pPrestin expression, we next evaluated if pPrestin expression could be further improved by extending the duration of sonication. Figure 10 demonstrates that the level and area of pPrestin expression could be efficiently improved by adjusting the time of sonication (60 s: 2300.5±465.9 a.u.; 120 s: 4893.9±785.3 a.u.; and 240 s: 8643.2±587.0 a.u.; the residual time of pPrestin-MBs *in vivo* less than 700 s, Fig. S5). As expected, we did not observe fluorescence signals in the contralateral brain (60 s: 100.5± 07.2 a.u.; 120 s: 80.5±39.7 a.u.; and 240 s: 70.5±22.5 a.u.).

### *In vivo* selective activation of transfected cells by 0.5-MHz US

**Figure 11:**
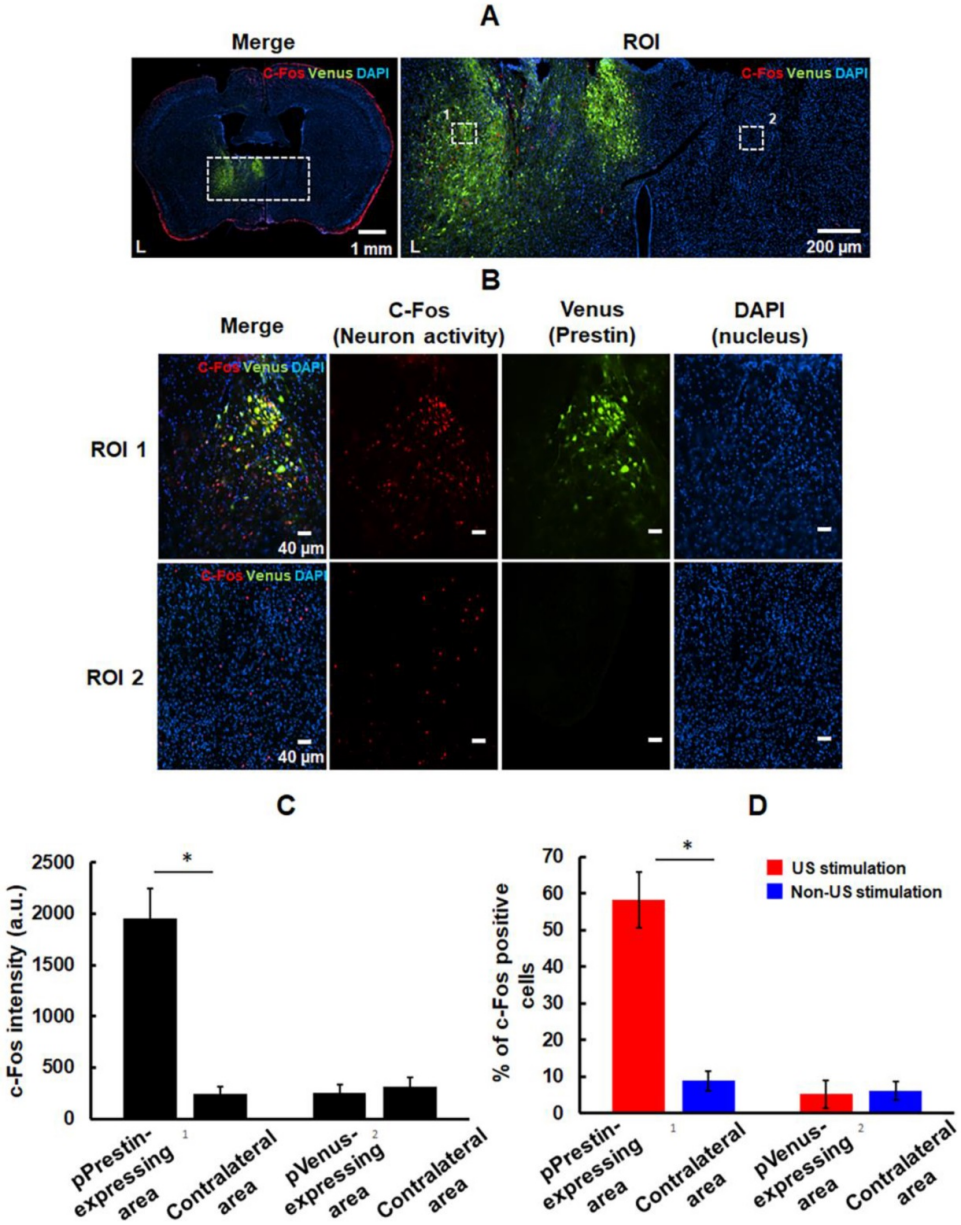
(A) Left: immunostaining for activation of pPrestin-transfected area and non-pPrestin-transfected area by 0.5-MHz US; right: Magnified view of ROI. (B) Local magnified view from ROI (white dot rectangle). (C) Quantification of c-Fos expression based on the intensity of red fluorescence protein in the region of interest. (D) Quantification of activated (c-Fos-positive) pPrestin-positive cells or pVenus-positive cells after 0.5-MHz US stimulation, compared to non-sonicated group. *: *p*<0.05. Data are shown as the mean ± standard deviation for 5-9 different sections (n = 4 per group).

We examined the feasibility of utilizing low-frequency (0.5-MHz) US to target the pPrestin-transfected cells. US-activated cells were mapped by imaging the expression of c-Fos. Note that the contralateral brain without pPrestin transfection also received 0.5-MHz US sonication for comparison. Compared to the area without pPrestin expression, c-Fos signals were detected in the pPrestin-transfected area following 0.5-MHz US stimulation, suggesting the excitation of neuronal activity (Fig. 11A-B) (pPrestin-transfected area: 1956.2±284.9 a.u. vs. non pPrestin-transfected area: 257.5±73.7 a.u.) (Fig. 11C). Venus alone expression group showed no significant increase in c-Fos expression after US stimulation (pVenus-transfected area: 241.1±70.3 a.u. vs. non pVenus-transfected area: 312.6±88.6 a.u.) (Fig. 11C; Fig. S6). We also compared the number of c-Fos-positive cells in the pPrestin or pVenus-expressing area with and without 0.5-MHz US stimulation. Figure 11D demonstrates a significant increase of c-Fos-positive neurons was observed after US stimulation in the pPrestin-expressing area (US stimulation group: 58.2±7.7 % vs. non-US stimulation group: 5.2±3.8 %). The Venus-expressing area showed no significant increase in c-Fos expression with and without US stimulation (US stimulation group: 8.8±2.7 % vs. non-US stimulation group: 6.1±2.5 %). We did not detect activated microglia in the region with pPrestin expression after US stimulation (Fig. S7), demonstrating that pPrestin-mediated sonogenetics is a safe, flexible, and non-invasive approach for sonogenetic control of neuronal activity.

## Discussion

Herein, we successfully combined ultrasonic opening of the BBB with MBs loaded with an engineered ultrasound-sensing transmembrane protein to transfect cells at specific locations in the brain so that the cells will respond to transcranial ultrasound stimulation. Our results demonstrate a paradigm for achieving gene transfection and neuromodulation through non-invasive methods. This paradigm provides many benefits over standing techniques for medical and research applications. Compared to intracranial injections for viral gene delivery, which are invasive and often require multiple injections to cover the target region, we proposed an approach that enables comprehensive and non-invasive transfection of an entire brain region in a single procedure and potentially can be translated to larger animals and humans.

Compared to existing ultrasonic neuromodulation strategies in which US directly activates or suppresses target neurons or releases local neuromodulatory compounds at local brain area, our technique can be used to stimulate specific cells without affecting other cells within the ultrasound- targeted site. The low spatial resolution of transcranial ultrasound (< 1 MHz), from several hundreds of micrometers to a few millimeters, means that different types of neurons might be stimulated during onset of ultrasound. This scenario might decrease the treatment efficiency and evoke unpredictable neuromodulation. Our technique could selectively stimulate ultrasound-sensitize neurons in a treatment area, resulting in precise neuromodulation.

The *in vivo* gene transfection data suggest that the transgene expression was markedly decreased within 7 days, which is consistent with findings from previous studies 35. This transient expression might be caused by delivered pDNA-induced immunostimulation. The pDNA is derived from bacteria and has CpG dinucleotides, and the DNA is predominantly unmethylated, whereas in mammalian DNA the frequency of CpG dinucleotides is mostly inhibited. This difference might evoke an inflammatory response of the host to eliminate the transfected cells. Previous studies also pointed out that the CpG motifs within the pDNA vector would activate proinflammatory cytokines via cationic lipid-pDNA complexes in the lung 36. To further prolong the duration of transgene expression, reducing the CpG content of the vector or administering antibodies to the cytokines during gene transfection seems to be a potential solution 37.

Until now, different classes of mechanosensitive ion channels have been shown to convert ultrasound stimuli into biological signals as a sonogenetic tool to manipulate the activities of neurons or other cells, including Piezo, MscL, and TRP-4 10-12. Heterologous expression of these proteins forms pore-like structures in the lipid bilayer of the target cell. Deformation of the cell surface opens the central pore and allows ion permeation, enabling cell sensing mechanical forces from the extracellular side 38-40. Prestin had been identified as a transmembrane protein residing in the outer hair cells (OHCs), but it is not an ion channel 41. Although we observed ultrasound-evoked Ca^2+^ signal in Prestin-expressing cells, the mechanism of Ca^2+^ influx through ultrasound-activated Prestin-expressing cells still needs further investigation. Because prestin acts as a piezoelectric amplifier to enhance the electromotile response in OHCs and mammalian cell lines 41, we suggest that the US-induced intramembrane bio-piezoelectric perturbation may be amplified by pPrestin and then trigger the observed calcium influx.

Recent studies have demonstrated that *in vivo* (in guinea pigs and mice) US stimulation may work via direct activation of auditory cortex neurons and indirectly stimulation of cortices around the auditory cortex (somatosensory and visual cortices) 42, 43. In the current study, our US stimulation (0.5 MHz, 3 s duration, 10 Hz PRF, 2000 cycles, 0.5 MPa) significantly activates Prestin-positive cells, but not neurons in the anterior auditory field, indicating the specificity of neuromodulation (Fig. S8(A)). These data indeed differ with the observations of previous studies. The major reason for this is that the acoustic energy used in these two studies was higher than that in the current study (30% duty cycle vs. 4% duty cycle). We also tested whether repeated auditory stimulation (0.5 MHz, 0.5 MPa, 1000 Hz PRF, 150 cycles, 6 s duration) causes increased c-Fos expression in mouse brains. Consistent with previous studies, the repeated auditory stimulation robustly increased c-Fos-positive cells both in auditory cortical regions and cells expressing Prestin (Fig. S8(A)). However, compared to our condition (0.5 MHz, 0.5 MPa, 10 Hz PRF, 2000 cycles, 3 s duration), the US of higher PRF and longer duration (0.5 MHz, 0.5 MPa, 1000 Hz PRF, 150 cycles, 6 s duration) could not further improve the success rate of neuromodulation (low: 58.1 ± 7.6; high: 49.1 ± 10.3%; Fig. S7B). In summary, the US condition used in this study can be used to specifically activate pPrestin-transfected cells with limited induction in non-transfected cells. These results clearly confirmed that our sonogenetic tools directly and specifically activate the cells that are genetically modified to express Prestin.

In this work, we used a novel ultrasound-based method with the ultrasound-sensing protein, Prestin, to selectively actuate cellular activity. Since excitation of pPrestin-MBs can be used for non-invasive local gene delivery and ultrasound can penetrate into deep tissue with low attenuation, we envision that this platform can be used to express Prestin in neurons from different portions of the brain for metabolic regulation or disease treatment. For instance, this could be used to target hypothalamic neurons that regulate blood glucose and feeding; activating glucose-sensing neurons in this area would regulate plasma glucagon and glucose, thus stimulating feeding and lowering insulin levels 44. Further, dopaminergic neurons in the midbrain that regulate intracerebral dopamine concentration have been studied broadly in models of reward, addiction, and Parkinson's disease 45-47. Modulation of dopaminergic neuronal activity may affect behavior or eliminate disease. The US with Prestin-induced extracellular Ca^2+^ influx could be used to actuate calcium-sensitive phosphatase calcineurin to dephosphorylate a transcription factor, the nuclear factor of activated T-cells (NFAT), which can trigger the expression of a target gene 48.

Our proposed strategy could be made more powerful with improvements across all components: ultrasound-sensing transmembrane (Prestin), MB-based gene vectors, and ultrasound stimulation conditions. For instance, the ultrasound sensitivity of Prestin can be improved using an engineered or mutated Prestin structure. Further work is required to design a more efficient MB-based gene vector to reduce the required dose, the DNA payload, and to make compact cell-type specific ligands to perform targeted gene transfection, including: (1) increasing neuronal targeting by changing the promoter of the Prestin plasmid (e.g., synapsin I promoter for neuronal cell; tyrosine hydroxylase (TH) promoter for dopaminergic neuron; vesicular glutamate transporter-1 promoter for glutamatergic neuron) 49-51; (2) increasing the targeting ability of MBs by modifying targeting ligand on to the surface of pPresin-MBs (e.g., fragment of tetanus toxin for neuronal cell; dopamine for doapminigic neuron) 52, 53. The feasibility of transfecting pPrestin via pPrestin-MBs with US into primary neurons and then exciting the pPrestin-expressing neurons by US also should be investigated. The ultrasound parameters for Prestin activation also need further optimization, including frequency, pressure, and cycle number. Finally, the c-Fos signals might be unreliable as a single measurement of activation. To further strengthen our conclusions, the future work of this study will use local field potential technique to evaluate the electrophysiology of neurons during ultrasound sonication 54. We propose that these modifications will enable us to achieve precise non-invasive regulation of neural circuits within the brain.

## Conclusions

In this study, we prepared ultrasound-sensing DNA (pPrestin)-loaded MBs for US-based non-invasive targeted gene delivery in the brain. Our results demonstrate that the ultrasound sensitivity of specific neurons can be enhanced by using this sonogenetic approach, improving the accuracy of conventional ultrasound-induced neuromodulation. Although the duration of transgene expression needs to be prolonged (currently less than 7 days), this approach provides a new perspective for research in brain neuroscience and for developing new tools for treating brain disorders such as Parkinson's disease and epilepsy.

## Supplementary Material

Supplementary figures.

Supplementary movie.
