# Supplementary material for: Targeted delivery of engineered auditory sensing protein for ultrasound neuromodulation in the brain
Source: Theranostics. 2020 Feb 18;10(8):3546–61. doi: 10.7150/thno.39786 (PMC7069068; doi:10.7150/thno.39786)
Supplement: Supplementary file 1 — Supplementary figures. [file thnov10p3546s1.pdf]

SUPPLEMENTARY MATERIALS

Targeted delivery of engineered auditory sensing protein for ultrasound neuromodulation in the brain

Chun-Yao Wu [a,1], Ching-Hsiang Fan [a,1], Nai-Hua Chiu [b], Yi-Ju Ho [a], Yu-Chun Lin [c,d,2] and Chih-Kuang Yeh [a,b,2]

[a] Department of Biomedical Engineering and Environmental Sciences, National Tsing Hua University, Hsinchu, Taiwan

[b] Institute of Nuclear Engineering and Sciences, National Tsing Hua University, Hsinchu, Taiwan

[c] Institute of Molecular Medicine, National Tsing Hua University, Hsinchu, Taiwan

[d] Department of Molecular Medicine, National Tsing Hua University, Hsinchu, Taiwan

[1] C.-Y. Wu and C.-H. Fan contributed equally to this work.

[2] Corresponding authors at: Department of Biomedical Engineering and Environmental Sciences, National Tsing Hua University, No. 101, Section 2, Kuang-Fu Road, Hsinchu, Taiwan 30013, R.O.C. Tel: +886-3-571-5131, ext. 34234; Fax: +886-3-571-8649 (C.-K. Yeh). Institute of Molecular Medicine, National Tsing Hua University, No. 101,

Section 2, Kuang-Fu Road, Hsinchu, Taiwan 30013, R.O.C. Tel: +886-3-574-2421 (Y.-C. Lin).

E-mail address: ckyeh@mx.nthu.edu.tw (C.-K. Yeh), ycl@life.nthu.edu.tw (Y.-C. Lin).

Table of Contents

Method

Immunostaining

VIDEOS

Movie S1. Calcium imaging of SH-SY5Y cells with or without pPrestin expression in response to 0.5-MHz US stimulation.

Figure

Figure S1. Time course of R-GECO fluorescence intensity in SH-SY5Y cells expressing pPrestin in the absence of 0.5-MHz US stimulation.

Figure S2. Cell experiments to verify pPrestin induced calcium influx from the extracellular space instead of from the intracellular calcium pool after US excitation.

Figure S3. The viability of SH-SY5Y cells expressing pVenus or pPrestin were stimulated by US or without US.

Figure S4. Immunostaining imaging to reveal what type of cell was transfected pPrestin.

Figure S5. The residual time of pPrestin-MBs *in vivo* measured by ultrasound B-mode imaging.

Figure S6. Immunostaining for activation of pVenus-transfected area and non pVenus-

transfected area by 0.5-MHz US.

Figure S7. US stimulation does not activate microglia in region with pPrestin expression.

Figure S8. Representative images of mice brain sections for verifying if US directly stimulated auditory cortex would elicit c-Fos signals in pPresin-expressing area.

Method

Immunostaining

The brains were then sliced into 15-μm sections and incubated into 5% goat serum and PBS for 1 h to block the endogenous proteins. The sections were then incubated in primary rabbit anti-Iba1 antibody (1:1000; Abcam, MA, USA), anti-VGLU2 antibody (1:1000; Abcam), anti-GFAP antibody (1:1000; Abcam), or anti-TH antibody (1:1000; Abcam) in antibody diluent for overnight. The sections were then incubated for 1 h in Dylight 594 conjugated anti-rabbit secondary antibody (1:500; Genetex) in antibody diluent followed by several washes in PBS. The cellular nuclei were labeled by DAPI. Finally, the slides were coverslipped with fluorescent mounting medium and stored flat in the dark at -20 °C.

To induced intracerebral hemorrhage, the mouse was injected self-made lipid-shell microbubbles solution (0.7-1.1 μm, 100 μL, 1×10^8 MB/mL) by retro-orbital injection. Twenty seconds later, the 0.5-MHz focused US (0.5 MPa, 10 Hz PRF, 5000 cycles, 120 s duration) was delivered to the left brain of mouse.

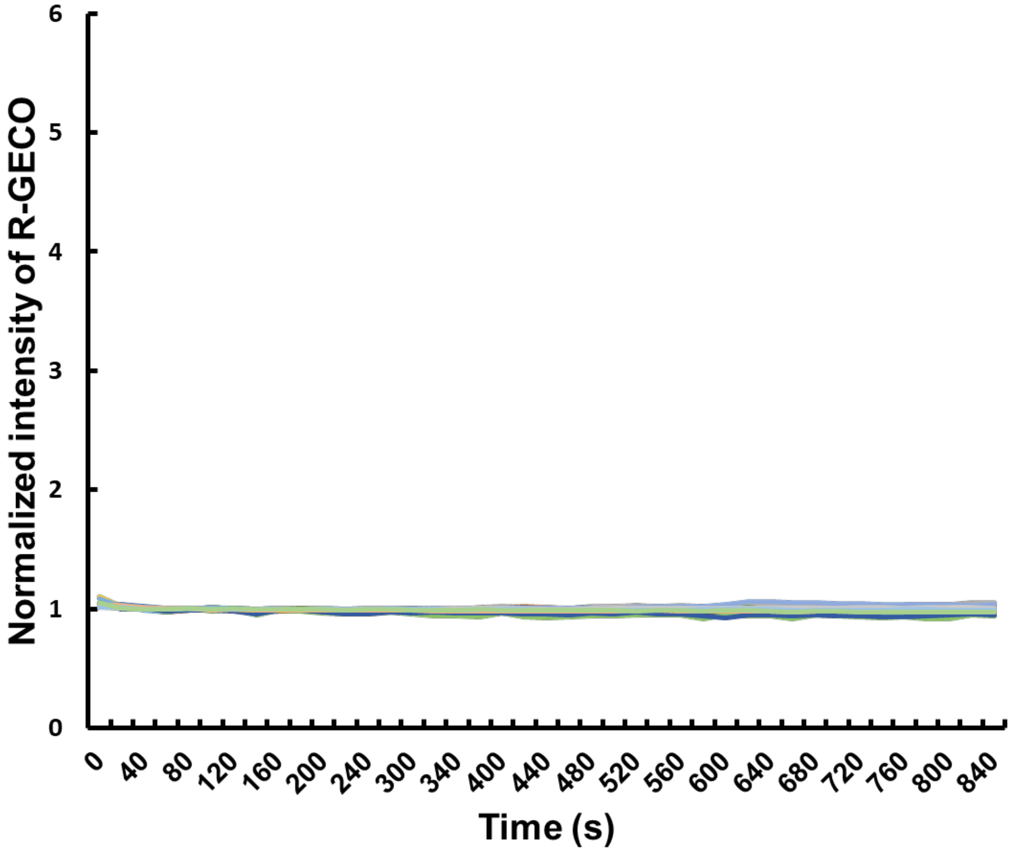

Figure S1. Time course of R-GECO fluorescence intensity in SH-SY5Y cells expressing pPrestin in the absence of 0.5-MHz US stimulation. Data were collected for 3 independent experiments.

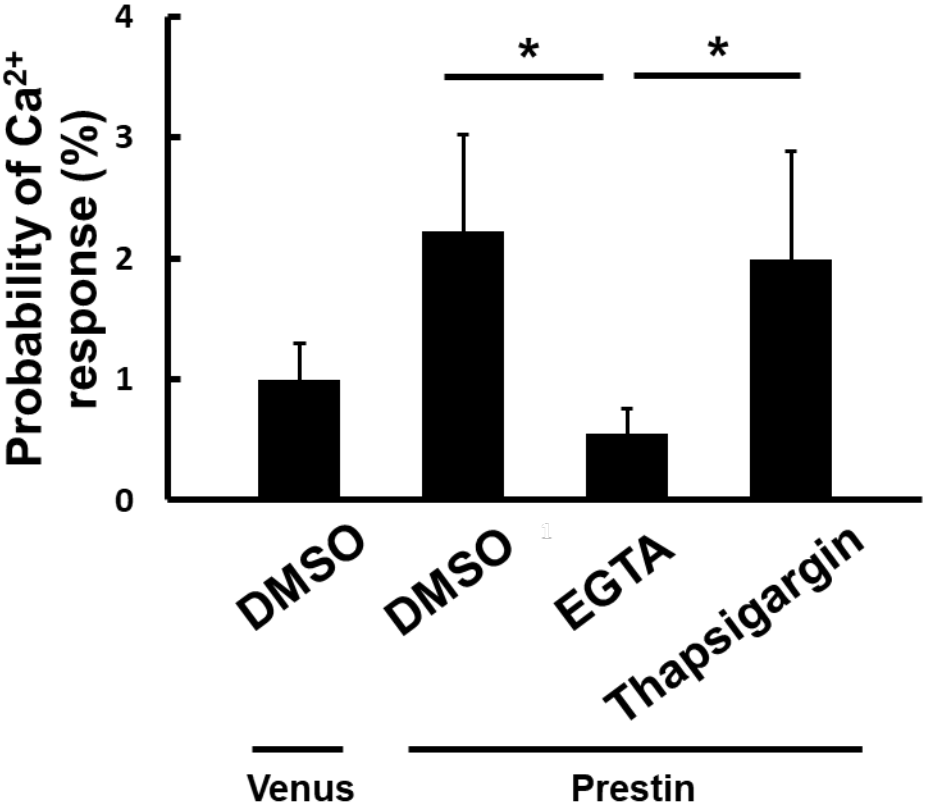

Figure S2. Cell experiments to verify pPrestin induced calcium influx from the extracellular space instead of from the intracellular calcium pool after US excitation. SH-SY5Y cells expressing pPrestin were incubated with ethylene glycol tetraacetic acid (EGTA, a calcium chelator in the extracellular space, 5 mM, 20 min) [1], or thapsigargin (depletion of the intracellular calcium store, 100 nM, 30 min) [2] in DMEM and were stimulated with 0.5 MHz US (0.5 MPa, 2000 cycles, 10 Hz PRF, 3 s duration); 0.1% DMSO served as the control. Data are shown as the mean ± standard deviation for 6 independent experiments.

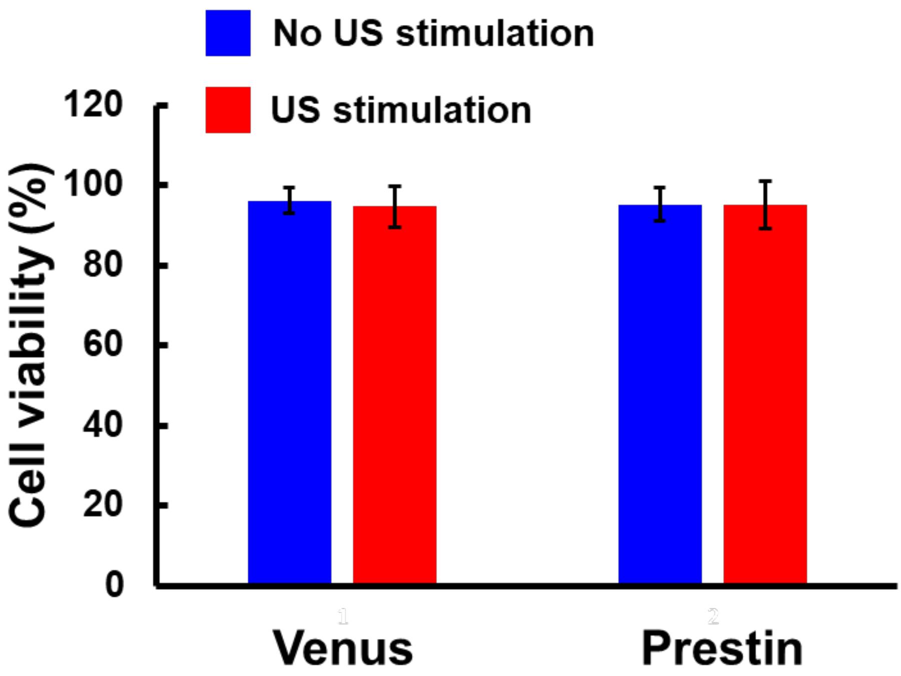

Figure S3. The viability of SH-SY5Y cells expressing pVenus or pPrestin were stimulated by US or without US. Data are shown as the mean ± standard deviation for 6–12 independent experiments.

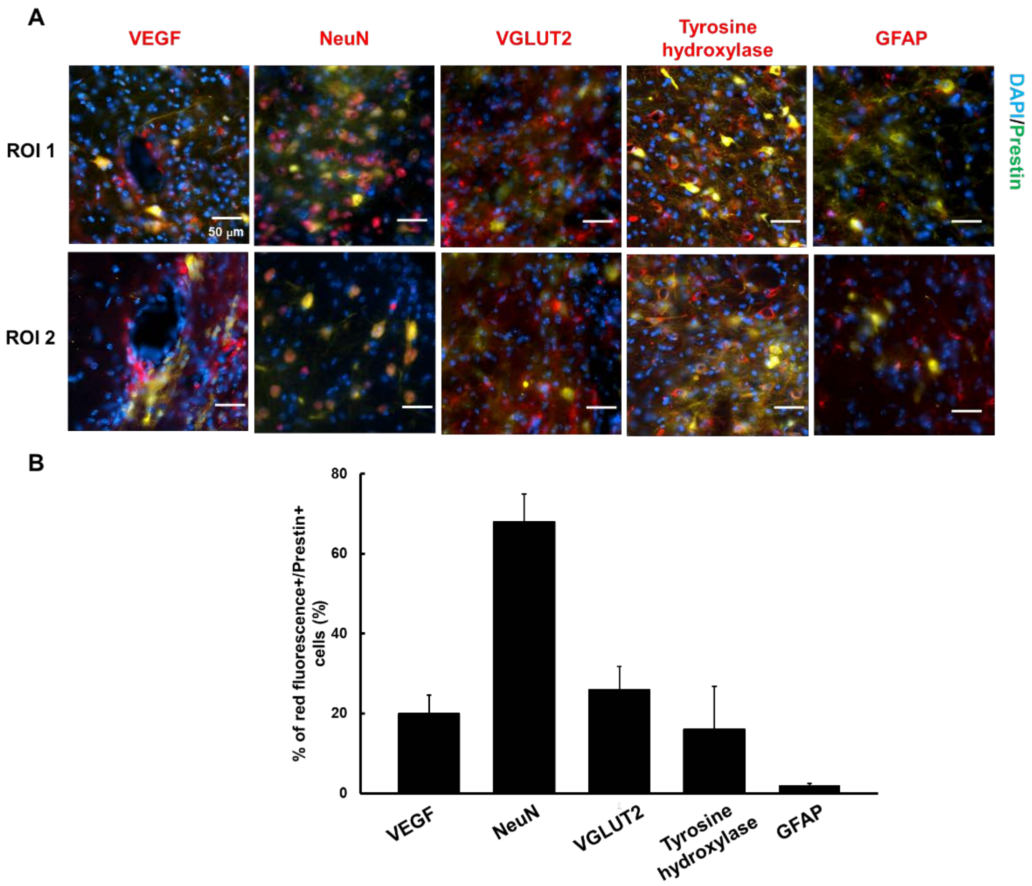

Figure S4. Immunostaining imaging to reveal what type of cell was transfected pPrestin. (A) Representative images of mouse brain sections after administration of pPresin-MBs and immunostaining with anti-vascular endothelial growth factor antibody (VEGF, a vascular endothelial cell marker), anti-NeuN antibody (a neuron marker), anti-VGluT2 antibody (a glutamatergic neuron marker), anti-tyrosine hydroxylase antibody (a dopaminergic neuron marker), or anti-GFAP antibody (an astrocyte marker), respectively. (B) Percentage of red fluorescence positive cells expressing pPrestin. The pPrestin expression was detected in VEGF-positive cells and in NeuN-positive cells after gene transfection. For neuron cells, the prestin expression was detected mainly in Tyrosine hydroxylase-positive neurons and rarely in VGlUT2-positive glutamatergic

neurons. Data are shown as the mean ± standard deviation for 5-9 different sections from 3 mice.

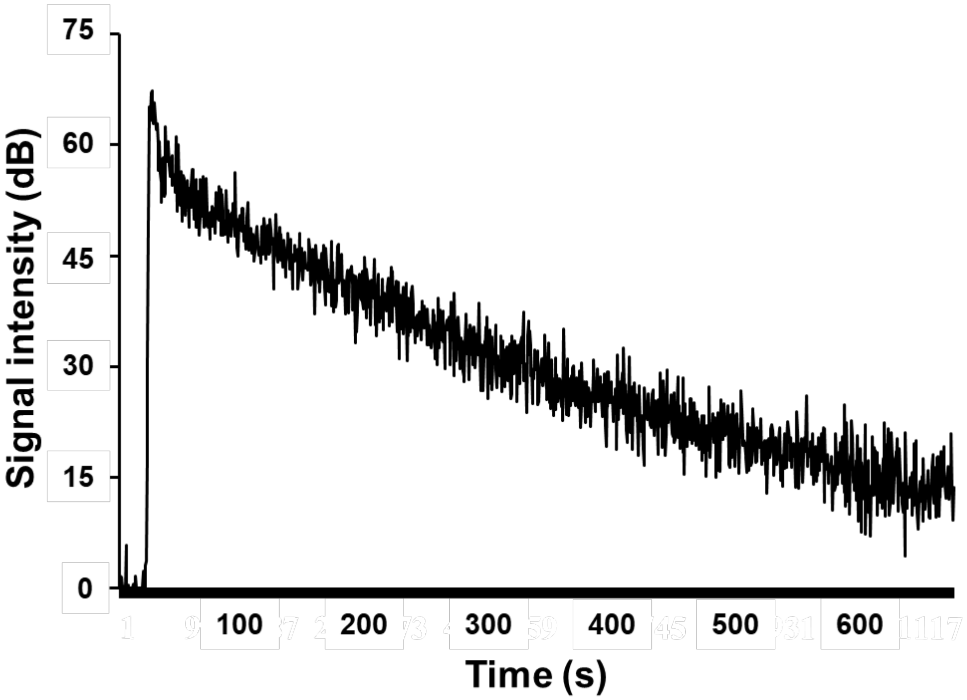

Figure S5. The intracerebral residual time of pPrestin-MBs *in vivo* was measured from ultrasound B-mode imaging by a 25-MHz ultrasound imaging system [3].

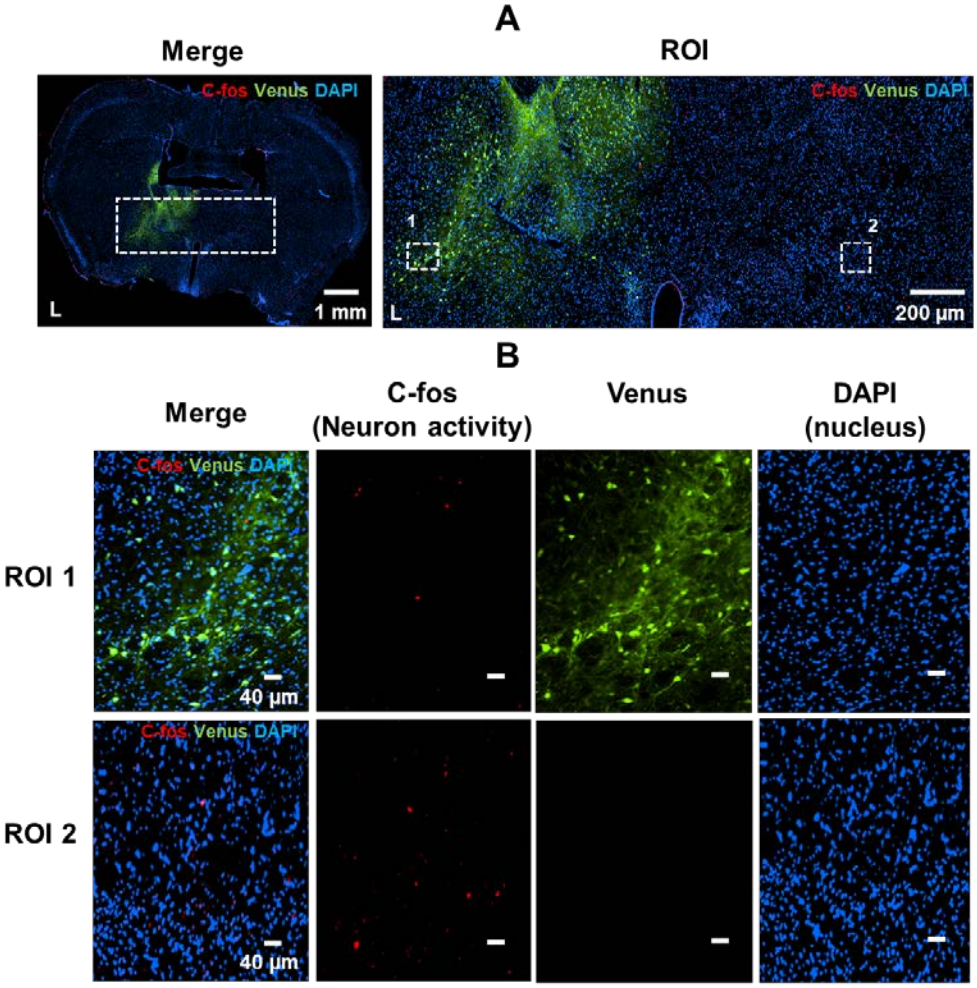

Figure S6. (A) Left: immunostaining for activation of pVenus-transfected area and non pVenus-transfected area by 0.5-MHz US; right: Magnified view of ROI. (B) Local magnified view from ROI 1 and ROI 2. pVenus was transfected by pVenus-loaded MBs with 1-MHz focused US (0.5 MPa) was used for gene delivery to the mouse brain at 2 days post-transfection with sonication times of 240 s.

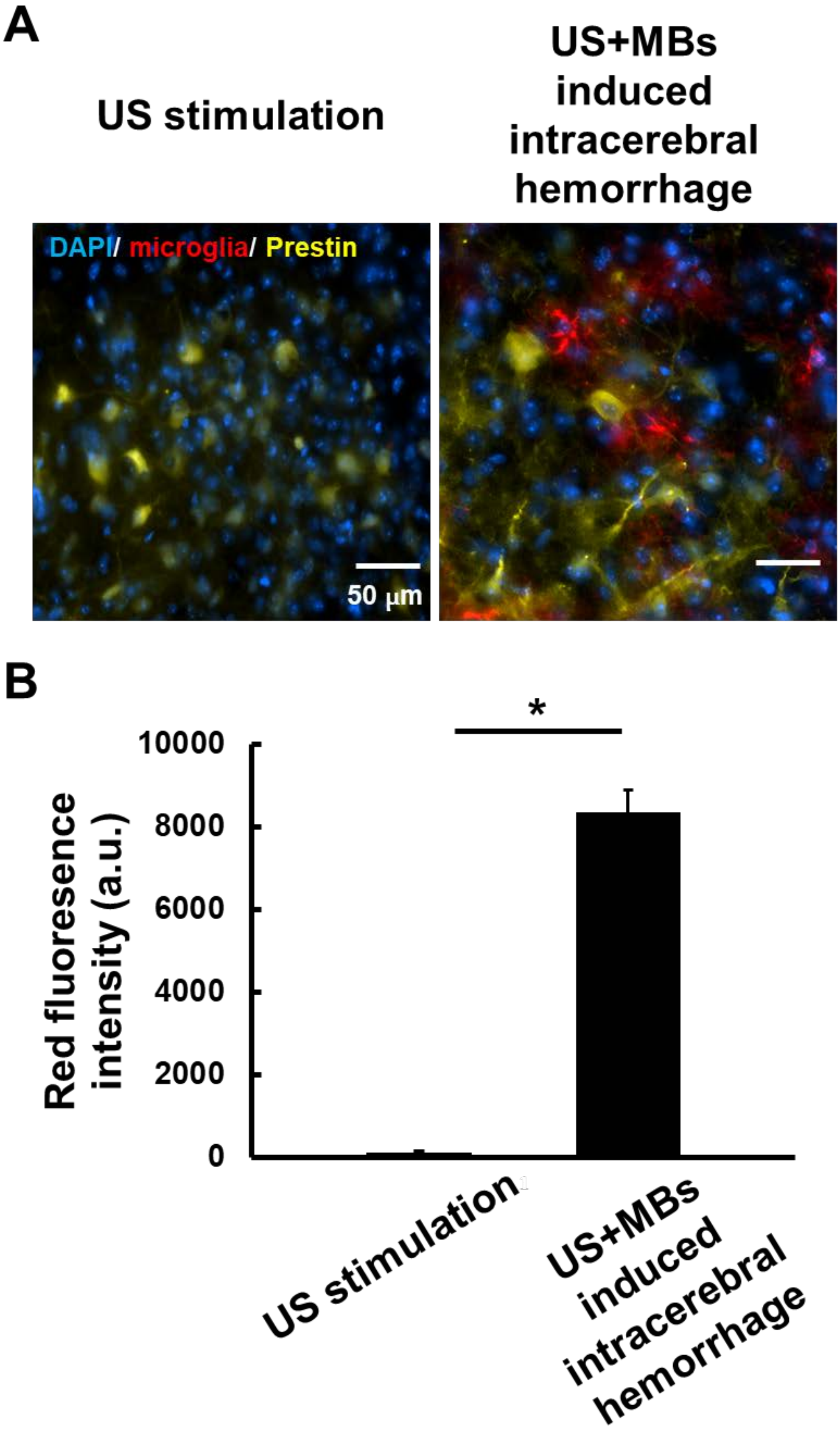

Figure S7. US stimulation does not activate microglia in region with pPrestin

expression. Representative images of mouse brain sections after US stimulation were immunostained with anti-lba1 antibody (a microglia marker). The parameters of US used to stimulate Prestin-positive cells are 0.5 MHz, 0.5 MPa, 10 Hz PRF, 2000 cycles, and 3 s duration. US+MBs induced intracerebral hemorrhage (1 MHz, 0.7 MPa, 1 Hz PRF, 5000 cycles, and 60 s duration, I.V. injected MBs) serves as a positive control of microglia staining. Data are shown as the mean ± standard deviation for 5-9 different sections from 3 mice.

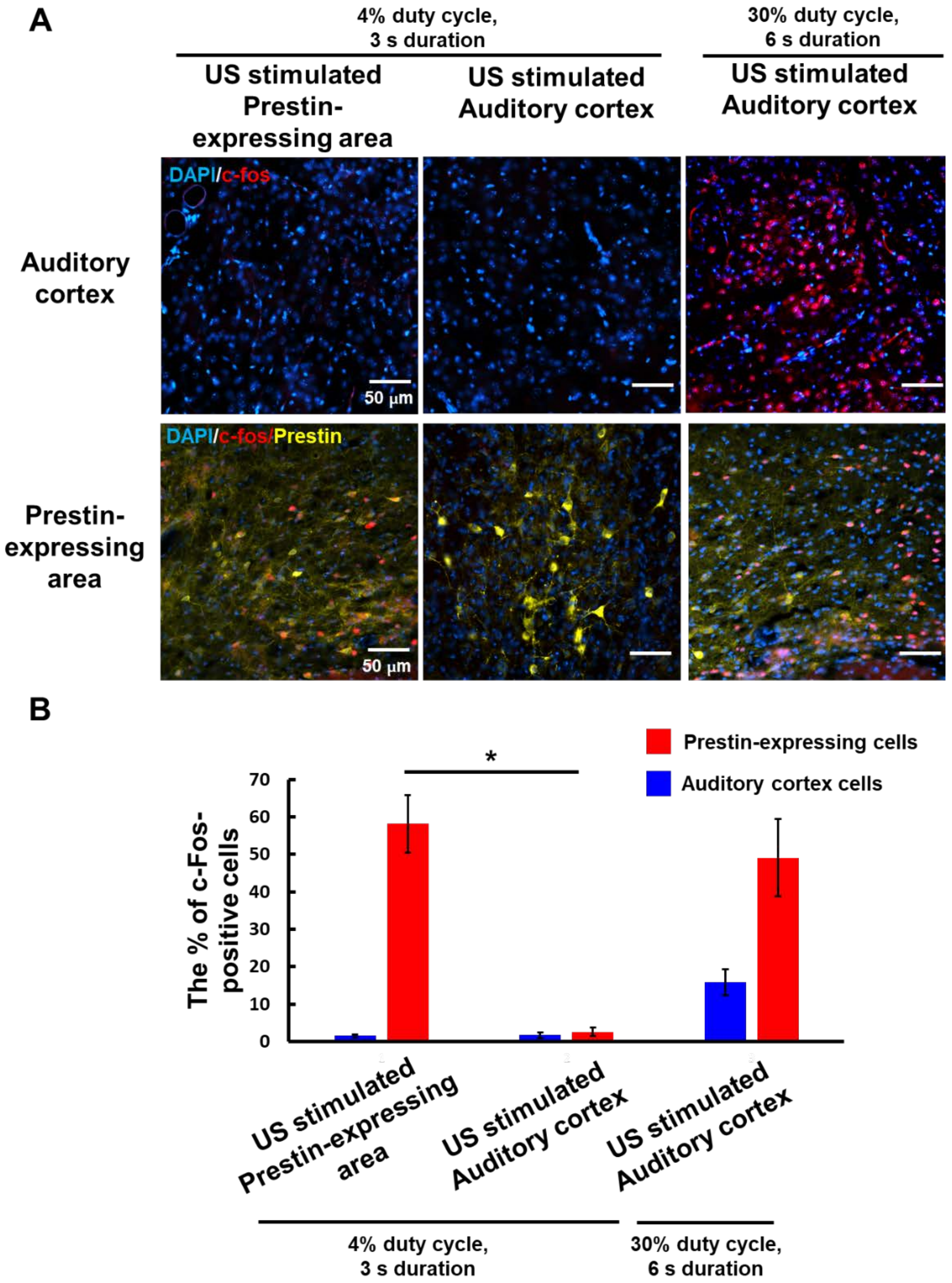

Figure S8. (A) Representative images of mice brain sections for verifying if US directly stimulated auditory cortex would elicit c-Fos signals in pPresin-expressing area. Top: brain sections of auditory cortex; bottom: brain sections of pPresin-expressing area. (B) Percentage of c-Fos-positive cells expressing pPrestin with different conditions. 4%

duty cycle: 0.5 MHz, 0.5 MPa, 10 Hz PRF, 2000 cycles, 3 s duration; 30% duty cycle: 0.5 MHz, 0.5 MPa, 1000 Hz PRF, 150 cycles, 6 s duration. The localized of auditory was according to a previous study [4]. Data are shown as the mean ± standard deviation for 5-9 different sections from 3 mice per condition.

Reference

[1] Marino A, Arai S, Hou Y, Sinibaldi E, Pellegrino M, Chang YT, et al. Piezoelectric Nanoparticle-Assisted Wireless Neuronal Stimulation. ACS Nano. 2015; 9: 7678-89.

[2] Engedal N, Torgersen ML, Guldvik IJ, Barfeld SJ, Bakula D, Sætre F, et al. Modulation of intracellular calcium homeostasis blocks autophagosome formation. Autophagy. 2013; 9: 1475-90.

[4] Ting C-Y, Fan C-H, Liu H-L, Huang C-Y, Hsieh H-Y, Yen T-C, et al. Concurrent blood-brain barrier opening and local drug delivery using drug-carrying microbubbles and focused ultrasound for brain glioma treatment. Biomaterials. 2012; 33: 704-12.

[4] Tsukano H, Horie M, Hishida R, Takahashi K, Takebayashi H, Shibuki K. Quantitative map of multiple auditory cortical regions with a stereotaxic fine-scale atlas of the mouse brain. Sci Rep. 2016; 6: 22315.
